# Fish consumption and resilience to depression in Japanese company workers: a cross-sectional study

**DOI:** 10.1186/s12944-015-0048-8

**Published:** 2015-05-26

**Authors:** Eisho Yoshikawa, Daisuke Nishi, Yutaka Matsuoka

**Affiliations:** Department of Neuropsychiatry, Nippon Medical School Tama Nagayama Hospital, Tokyo, Japan; Department of Neuropsychiatry, Nippon Medical School, Tokyo, Japan; Department of Psychiatry, National Disaster Medical Center, Tokyo, Japan

**Keywords:** Long chain n-3 poly unsaturated fatty acids, Fish consumption, Depression, Resilience

## Abstract

**Background:**

Depression is a common disorder that is influenced by psychosocial factors in the workplace. Increasing resilience, the ability to cope with stress in the face of adversity, is considered an important strategy to prevent depression. It has been suggested that consumption of fish, which is a major source of long chain n-3 polyunsaturated fatty acids (LC n-3 PUFA), may prevent depression. However, associations between depression, resilience, and fish consumption have not been documented.

The aim of the study is to investigate the association between fish consumption and resilience to depression.

**Methods:**

Participants were 527 Japanese employees at three worksites of a large company. The Center for Epidemiologic Studies Depression (CES-D) Scale was administered to assess depressive symptoms, and the 14-item Resilience Scale (RS-14) was administered to assess resilience. A self-report questionnaire extracted from the Food Frequency Questionnaire was used to measure fish consumption frequency. Regression analyses were conducted to assess a mediation model based on a statistical analysis framework defined by Baron and Kenny. The indirect association of resilience was calculated with the bootstrapping method. Each analysis was adjusted by age, sex, marital status, work position, and educational background.

**Results:**

The association between fish consumption frequency and total CES-D score was significant (B = −0.94; *p* = 0.011). The association between fish consumption frequency and total RS-14 score was significant (B = 1.4; *p* = 0.010), as was association total RS-14 score and the total CES-D score (B = −0.34; *p* < 0.001). When controlling for total RS-14 score, there was no longer a significant association between fish consumption frequency and total CES-D score. The bootstrapping results revealed that significant indirect association though fish consumption frequency and total CES-D score (bias corrected and accelerated confidence interval = −0.83 to −0.13; 95 % confidence interval) through total RS-14 score.

**Conclusions:**

Fish consumption might be associated with resilience to depression. Further studies are needed, particularly double blind randomized placebo controlled intervention trials on the potential preventative effect of LC n-3 PUFA on resilience to depression.

## Background

Depression is a common disorder in the workplace and can result in impaired job performance [1], long absences due to sickness [2], and the need to pay disability pension [3]. The resulting loss due to reduced work capacity has been estimated to account for around one working month per ill worker per year [4]. Depression therefore represents a measurable economic burden to society [5, 6]. Adverse psychosocial factors, such as job strain (high demand and low decision latitude in the workplace) may be related to an elevated risk of subsequent depressive symptoms or major depressive episodes [7], and effective prevention strategies against depression are particularly important to both employees and employers, as well as society as a whole.

The attribute of resilience has garnered considerable attention in efforts to prevent mental disorders. Resilience is defined as a dynamic process of adaptation to challenging life conditions [8, 9], and it has generally been viewed as the ability to cope with stress in the face of adversity [10]. Resilient persons tend to manifest adaptive behaviors [8], experience positive emotions even in stressful situations [11], and express emotional flexibility naturally in response to rapidly changing events [12]. Resilience has been associated with regulation of emotions and negatively associated with depression [11, 13]. Increasing resilience is therefore viewed to be an important strategy to prevent depression [14].

Epidemiological evidence has shown a negative association between depression and fish consumption. For example, Hibbeln found a strong negative association between the two factors in 13 countries [15], and Tanskanen et al. found a higher prevalence of depression in low fish consumers than in high fish consumers in Finland [16]. Timosen et al. reported a higher risk of depression in women who ate fish rarely compared with those who ate fish more frequently [17]. Fish is rich source of long chain n-3 polyunsaturated fatty acids (LC n-3 PUFA), namely eicosapentaenoic acid (EPA) and docosahexaenoic acid (DHA). In human, EPA and DHA can be synthesized from Alpha-linolenic acid (ALA), which is one of the two essential fatty acid that must be obtained from diets [18]. However the synthesis from ALA to DHA appears to be minimal [18] and there are evidences that DHA may be a semi-essential nutrient [19], which should be consumed directly from diet. There is high amount of LC n-3 PUFA in the brain and important structural component of the brain cell membrane. Parletta, et al. proposed six key mechanisms of the LC n-3 PUFA on the mental health as follow, 1) playing important role of neurite growth, 2) increasing fluidity and flexibility of cell membrane of the brain, 3) improving the function of neurotransmitter such as serotonergic and dopaminergic system 4) playing important role in endothelial function which may improve blood flow and blood brain barrier integrity via anti-inflammatory, vasodilatory, and increasing glucose transport 5) to encourage neuronal survival via increasing synthesis PS-DHA 6) to prevent neurodegeneration through increasing synthesis of neuroprotectin D1(NDP1) [20]. Therefore, LC n-3 PUFA may have important role for the regulation of mood and behavior. In addition, several meta-analyses have demonstrated the benefit of supplementation with LC n-3 PUFA for depression [21–24].

Given the above, frequent fish consumption may attenuate depressive symptoms by enhancing resilience. However, associations between fish consumption, resilience, and depression have not been well documented. The aim of this study was to investigate the association between fish consumption and resilience to depression.

## Results

### Participant demographics

All 527 participants were Japanese. The participants averaged 38.3 ± 9.0 years of age (range = 23 – 63), 426 (80.8 %) were men, and 318 (60.3 %) were married. Regarding educational background, 456 (85.5 %) had graduated from college or university. Regarding working status, 41 (7.8 %) were in management positions, 58 (11.0 %) held clerical jobs, 404 (76.7 %) were engineers, and 15 (2.7 %) performed skilled work. Mean scores + Standard Deviation on the three parameters assessed were 63.5 ± 11.0 on the 14-item Resilience Scale (RS-14), while Mean scores + Standard Deviation was 10.5 ± 7.5 on the Center for Epidemiologic Studies Depression (CES-D) Scale, and 3.0 ± 0.9 for the fish consumption score on the self-report questionnaire extracted from the Food Frequency Questionnaire (FFQ) on frequency of fish consumption. The score “3.0 ± 0.9” in the current study means that mean response of participants was 1–2 times per week of fish consumption, and mean - SD was 2.1 which meant about 1–3 times per month of fish consumption and mean + SD means was 3.9 which meant about 3–4 times per week of fish consumption.

### Univariate analysis

The results of the univariate analyses are shown in Tables 1 and 2. Among the significant findings were that men ate fish more frequently than women, and married people had lower CES-D scores, higher RS-14 scores, and ate more fish than unmarried people (Tables 1 and 2). Those in management positions had higher RS-14 scores than those in non-management positions. The fish consumption frequency was positively associated with age, total CESD score and total RS-14 score.

**Table 1:** Results of univariate analyses of the association of demographic categories on depression and resilience

			CES-D				RS-14			
			r	*p*			r	*p*		
Age			0.039	0.368			0.078	0.07		
		n	Mean	SD	*t*	*p*	Mean	SD	*t*	*p*
Sex	male	426	10.4	7.4	−0.74	0.462	63.7	11.2	0.84	0.399
	female	101	11.0	7.8			62.7	10.5		
Married	yes	318	9.4	6.8	−4.27	<0.001	65.1	10.4	4.26	<0.001
	no	209	12.3	8.2			61.0	11.5		
Graduated from university or college	yes	456	10.3	7.4	−1.71	0.089	63.5	1.3	−0.02	0.982
	no	71	11.9	8.0			63.5	1.5		
Management position	yes	41	8.8	6.2	−1.58	0.113	67.0	10.8	2.11	0.036
	no	486	10.7	7.6			63.2	11.0		

*CES-D* Center for Epidemiologic Studies Depression, *RS-14* 14-item Resilience Scale, *SD* standard deviation

**Table 2:** Results of univariate analyses of the association between demographic characteristics, depressive symptoms, resilience and fish consumption frequency

			Fish consumption frequency		
			rho	*p*	
Age			0.153	<0.001	
CES-D			−0.096	0.027	
RS-14			0.149	<0.001	
		n	mean	SD	*p*
Sex	male	426	3.0	0.9	0.014
	female	101	2.8	0.8	
Married	yes	318	3.1	0.8	<0.001
	no	209	2.8	0.9	
Graduated from university or college	yes	456	2.9	0.9	0.094
	no	71	3.1	1.0	
Management position	yes	41	2.9	0.9	0.952
	no	486	3.0	0.9	

*CES-D* Center for Epidemiologic Studies Depression, *RS-14* 14-item Resilience Scale, *SD* standard deviation

### Mediation analysis

The total mediator model was significant F (7, 519) = 30.45, *p* < 0.0001. The total explained variance (R^2^) was 29.1 %, and the adjusted R^2^ was 28.2 %. As shown in Table 3, in the bootstrap analysis, the association between fish consumption frequency and total CES-D score was significant (B = −0.94; *p* = 0.011). The association between fish consumption frequency and total RS-14 score was significant (B = 1.4; *p* = 0.010), as was association total RS-14 score and total CES-D score (B = −0.34; *p* < 0.001). When controlling for total RS-14 score, there was no longer a significant relationship between fish consumption frequency and total CES-D score. The bootstrapping results revealed that significant indirect association though fish consumption frequency and total CES-D score (bias corrected and accelerated confidence interval = −0.83 to −0.13; 95 % confidence interval) through total RS-14 score.

**Table 3:** Results of mediation analysis and bootstrapping

	B	SE	*t*	*p*	BCACI
a path	1.40^a^	0.54	2.57	0.010	-
b path	−0.34^a^	0.03	−13.07	<0.001	-
c' path	−0.47^a^	0.32	−1.47	0.142	-
c path	−0.94^a^	0.37	−2.57	0.011	-
(c – c') path	−0.47	0.18			−0.83 to −0.13

*SE* Standard error, *BCACI* the bias corrected and assessed confidence intervals

**Fig. 1:**
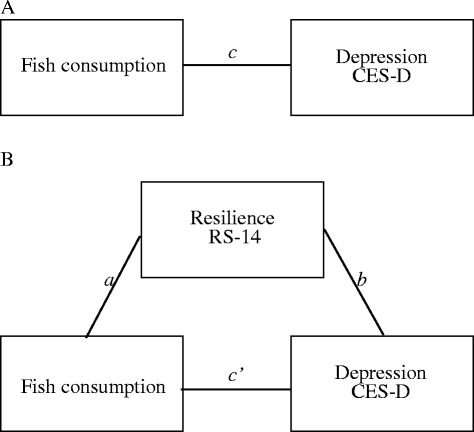
**a** Illustration of a direct association of the dietary factor of fish consumption on depression. Path *c* represents the total association of fish consumption on depression (CES-D). **b** Illustration of an indirect association between fish consumption and depression (CES-D) through resilience (RS-14). Path *a* represents the association between fish consumption and resilience (RS-14), the proposed mediator. Path *b* represents the association between resilience (RS-14) and depression (CES-D), without fish consumption. Path c′ is the association between the fish consumption and depression (CES-D), without resilience (RS-14). The indirect association between the fish consumption and depression (CES-D) through resilience (RS-14) score is c – *c′*, which is tested with the confidence interval obtained through the bootstrapping method

^a^Regression coefficient between the pair of variables at each end of the indicated path (see Fig. 1)

## Discussion

This is the study to investigate the association between fish consumption and resilience to depression. Participants in current study replied that they usually ate fish or fish meals such as raw fish and/or grilled fish about 1–2 times per week on average. In univariate analysis, the older, married people, and men consumed fish more frequently, while males are more predominant (80 %) in this study. The result of the study suggested that fish consumption significantly associated with resilience to depression. Additionally, the results as analyzed with the statistical framework defined by Baron and Kenny [25], suggested that indirect association between fish consumption and depression thorough resilience. To best our knowledge there were few studies to investigate the association between fish consumption and resilience to depression, except for a study by Matsuoka and Nishi investigated the relationship between resilience and dietary factors, but not with depression, and found a positive association between higher fish consumption and resilience among rescue workers after the Great East Japan Earthquake [26].

Several lines of evidence suggest an association between resilience and emotional regulation. Tugade and Fredrickson examined cardiovascular reactivity in a stressful task and demonstrated that participants with higher resilience recovered more quickly from the cardiovascular arousal than participants with lower resilience, and this faster recovery was mediated by positive emotions [11]. Waugh demonstrated that participants with higher resilience show more appropriate and flexible emotional regulation in response to an anticipated threat than those with lower resilience [12, 27]. A functional magnetic resonance imaging study indicated that when anticipating a threat, the anterior insula, a region associated with emotional regulation, was activated for a shorter time in highly resilient participants than in those with low resilience [28].

Furthermore, several studies have associated the regulation of emotion in stressful situation with n-3 PUFA intake. Rats that experienced an early stressful situation of maternal separation showed less capacity to control fear responses when they lacked dietary n-3 PUFA [29]. Hamazaki et al. reported that DHA intake by university students prevented aggression against others from increasing at times of mental stress during the students’ busiest and most frustrating days [30]. However, they did not find an effect on aggression in the absence of a stressful situation [31]. Suzuki et al. [32] found that intake of α-linolenic acid and total n-3 PUFA might be associated with decreased incidence of depression in response to newly diagnosed lung cancer. Chang et al. [33] found that moderate depression, not severe, with cardiovascular disease had lower levels of DHA, n3-PUFA, and n6/n3 ratio in erythrocyte membranes compared with non-depression, while there were no difference in the electrocardiac markers and inflammation marker. Nishi et al. reported that fish oil supplementation attenuated posttraumatic symptoms in female rescue workers after the Great East Japan Earthquake [34]. Hence, fish consumption might promote the biological basis of appropriate and flexible emotional regulation in stressful situations, thus enhancing resilience.

Several studies have also suggested a possible preventive effect of LC n-3 PUFA supplementation on bipolar disorder [35], psychotic disorder [36], posttraumatic stress disorder following accidental injury [37], and interferon α–induced depression [38]. Dietary modification is widely recognized and promoted for the primary prevention of non-communicable disorders, such as cardiovascular disease, obesity, and diabetes. Although strategies for preventing depression have traditionally focused on pharmacological and psychological approaches, O’Neil et al. recently designed a study to investigate the effect of an individualized, structured dietary intervention on depression [39]. In addition, several approaches that increase resilience, such as well-being therapy, are used to treat depression [14, 40], by not only attenuating and preventing negative symptoms but also promoting positive emotions in order to increase psychological well-being [14]. Such a psychotherapeutic approach may increase resilience and prevent recurrence of depression [41, 42]. The findings of current study cannot deny the possibility that fish consumption increase the resilience and prevent depression, but this warrants further investigation.

## Conclusion and limitations

Fish consumption might be associated with resilience to depression in Japanese company workers. However, further studies are needed, particularly double blind randomized placebo controlled intervention trials on the potential preventative effect of LC n-3 PUFA on resilience to depression.

Our study had several limitations. First, the participants were mainly men, they were highly educated, and they worked for a big Japanese company that provides good job security and a relatively good balance of effort and reward. Therefore, they might not be representative of workers more generally. Second, this study was conducted in Japan, a country already known to have very high levels of fish consumption, which is likely to have influenced LC n-3 PUFA levels in comparison with other populations. Third, information on fish intake frequency was self-reported, and non-differential misclassification may be inevitable and could attenuate the observed associations. Furthermore, specific food frequency questionnaire for LC n-3 PUFA intakes was not used in this study. It was reported that a specific food frequency questionnaire to assess LC n-3 PUFA intakes was superior to a generic FFQ [43]. Therefore, we may not be able to assess LC n-3 PUFA intakes in this study appropriately. In addition, no other dietary ingredients that might affect resilience were evaluated in this study. Fourth, due to the cross-sectional nature of the study design, causal relationships between the factors could not be determined. Finally, residual confounding by uncontrolled or unmeasured factors may have distorted genuine associations.

## Methods

### Participants and procedures

This study was approved by the institutional review boards of both the National Disaster Medical Center and the company. This study was conducted using data collected in a previous study [44]. A cross-sectional study was carried out at three separate worksites of a large company located in an urban area of Japan between August and November 2010. The inclusion criteria were as follows: company worker, age ≥ 18 years, and capable of understanding and providing consent for study participation. The company’s occupational health staff provided participants with a written explanation of the research, a consent form, and the self-reporting questionnaire. Workers who agreed to participate in this study provided consent by returning the consent form and questionnaire by postal mail.

Of the 6204 workers at the three sites of the company, 807 workers (13 %) were approached. Among them, 538 (66.7 %) agreed to participate in the study. We excluded 11 participants with missing responses to items related to the subscales used, leaving 527 participants for analysis in this study. The workers who did not participate did not differ significantly from the participants in terms of age or sex.

### Measures

#### Demographics

We gathered information on sex, marital status (married or not), educational background (graduated from college or university or not), and job status (management position or not).

#### Assessment of fish consumption in daily life

To evaluate fish consumption in daily life, we extracted one items from the short version of the FFQ [45]. Fish consumption was assessed with a frequency question: “How often do you usually eat fish or fish meals such as Sashimi (raw fish) and/or Yakizakana (grilled fish)? Please consider the last six months.” Six response options were given for each question: almost none, 1–3 times/month, 1–2 times/week, 3–4 times/week, 5–6 times/week, and every day.

#### Assessment of depressive symptoms

We administered the CES-D to assess depressive symptoms. This scale is one of the most widely used scales to assess depressive symptoms in the general population and measures the level of depressive symptoms in the past week [46]. The CES-D is composed of 20 items, and the scores are summed to yield a total score between 0 and 60, with a higher score indicating more severe depression. The reliability and validity of the Japanese version have been verified [47].

#### Assessment of resilience

The Resilience Scale (RS) is a self-reported questionnaire consisting of 25 items that measure the degree of individual resilience [8]. The RS was developed based on a qualitative study of 24 older women in America who had experienced a recent loss (e.g., of a spouse, health, or employment) and had adapted successfully [8, 48–52]. Its conceptual framework is composed of five characteristics. Items are categorized by (i) self-reliance (e.g. ‘I feel I can handle many things at a time’); (ii) meaning (e.g. ‘I feel proud that I have accomplished things in life’); (iii) equanimity (e.g. ‘I usually take things in stride’); (iv) perseverance (e.g. ‘I am determined’); and (v) existential aloneness (e.g. ‘my belief in myself gets me through hard times’). The shorter RS-14 version, consisting of 14 items, strongly correlates with the RS. Each item is rated on a 7-point Likert scale (range, 14–98), with a higher score indicating more resilience [8]. The reliability and validity of the Japanese version have been verified (10).

### Statistical analysis

#### Univariate analysis

Two-tailed Pearson’s correlations were used to examine the intercorrelation between age, total CES-D score, and total RS-14 score. Spearman’s rank correlation test was used to examine the intercorrelation between fish consumption frequency and age, total CESD score, and total RS-14 score. Student’s *t*-test was used to compare CES-D and RS-14 scores between demographic categories. The Mann–Whitney *U* test was used to compare fish consumption frequency between demographic categories.

#### Mediation analysis

We investigate the indirect association between the fish consumption frequency and total CESD score through the total RS-14 scores illustrated (Fig. 1a and b). Indirect association is assessed by comparing the total association (*c*) of an independent variable on a dependent variable, which is composed of a direct association (*c′*) of the independent variable on the dependent variable and an indirect association (*c* – *c′*) of the independent variable on the dependent variable through a proposed mediation variable. Coefficient *a* represents the association of the independent variable on the mediation variable M, whereas coefficient *b* represents the association of M on the dependent variable. According to the statistical analysis framework defined in [25], mediation models require that *a, b,* and *c* are significant and that *c′* is smaller than *c* by a nontrivial amount [53].

Three regression analysis were conducted to assess the mediation model in the statistical analysis framework defined by Baron and Kenny [25]. Each analysis was adjusted by age, sex, marital status, employment position, and educational background. The first regression analysis was conducted to evaluate the *c′* path, with total CES-D score as the dependent variable and fish consumption frequency as the independent variable. The second regression analysis was conducted to evaluate the *a* path, with total RS-14 score as the dependent variable and fish consumption frequency as the independent variable. The third regression analysis, which represented the total mediator model, was conducted to evaluate the *b* and *c* paths, with total CES-D score as the dependent variable and fish consumption frequency and total RS-14 score as independent variables.

Next, the indirect association was calculated from the unstandardized regression weights of paths *a* and *b* [25]. Bootstrapping was used to produce the sampling distributions of the indirect association by sampling from the data set (in this case, *n* = 5000 samples) and calculating the indirect association present in the resamples.

All of the analyses were performed using SPSS, version 21 (SPSS Inc., Chicago) and Preacher and Hayes’ bootstrap script for SPSS [54]. BCACI was set at 95 %. The use of a 95 % confidence interval is equivalent to testing for significance at the .05 level.

## Abbreviations

LC n-3 PUFA: Long chain n-3 polyunsaturated fatty acids
FFQ: Food Frequency Questionnaire
CES-D: The Center for Epidemiologic Studies Depression
RS-14: 14-item Resilience Scale
CI: Confidence interval
BCACI: Bias corrected and accelerated confidence interval
